# Acute cemented total hip arthroplasty combined with open reduction and internal fixation for elderly acetabular fractures: a two-center retrospective cohort study

**DOI:** 10.1007/s00068-026-03096-6

**Published:** 2026-02-23

**Authors:** Yasuhisa Ueda, Koichi Inokuchi, Yoshiaki Kurata, Makoto Sawano

**Affiliations:** 1 Department of Emergency and Critical Care Medicine, Saitama Medical Center, Saitama, Japan; 2 Division of Orthopaedic Trauma, Sapporo Tokushukai Hospital, Hokkaido, Japan

**Keywords:** Acetabular fracture, Cemented cup, Acute THA, Elderly, ORIF, Early mobilization

## Abstract

**Purpose:**

The aim of this study was to evaluate the outcomes of cemented total hip arthroplasty (C-THA) combined with open reduction and internal fixation (ORIF) in elderly patients with acetabular fractures, focusing on immediate postoperative stability and functional recovery.

**Methods:**

A retrospective cohort study was conducted on 51 patients with displaced acetabular fractures aged 60 years or older. Patients were divided into two groups: the C-THA group (*n* = 10) and the ORIF group (*n* = 41). Surgical procedures and outcomes, including time to full weight bearing (t-FWB), operative time, blood loss, Harris Hip Score (HHS at 3 months), poor prognostic factors (PPF), and complications were analyzed.

**Results:**

The C-THA group achieved significantly earlier weight-bearing (median 3.9 (2.8-5) vs. 70 (63–70) days, *p* < 0.001) and a trend toward lower blood loss (median 914 vs. 1300 ml, *p* = 0.060). PPFs were more common in the C-THA group (3.50 vs. 2.00, *p* = 0.025). While HHS at 3 months showed no statistically significant difference, trends favored C-THA. Bone union was achieved in all patients.

**Conclusion:**

C-THA may provide stable fixation allowing for immediate full weight-bearing and may be a preferable option in elderly patients with poor prognostic factors. Further long-term studies are needed to confirm these findings.

## Introduction

As the global population continues to age, the incidence of acetabular fractures among the elderly has increased significantly, and outcomes remain concerning. Reported 1-year mortality after geriatric acetabular fracture ranges from ~ 8% to 25%, with recent cohorts indicating ~ 21% in older patients, underscoring the frailty of this group and the clinical importance of enabling early mobilization [1, 2]. These fractures, often caused by low-energy falls, present substantial challenges in orthopaedic treatment due to poor bone quality from osteoporosis and pre-existing degenerative joint disease in this population [3]. Even with surgical fixation, the mechanical stability may be insufficient due to the fragility of the bone.

Traditionally, open reduction and internal fixation (ORIF) has been the mainstay of treatment; however, its efficacy in elderly patients is limited. The effectiveness of ORIF is diminished in the presence of poor bone stock or joint arthropathy, often resulting in unsatisfactory outcomes [4, 5]. Moreover, ORIF in elderly patients often entails a period of restricted or non-weight bearing, which is associated with increased medical complications and loss of function in this frail population [6]. This is a critical concern, as immobilization and delayed mobilizations have been linked to higher short- and mid-term mortality in orthogeriatric fracture patients [7].

To address these limitations, acute “fix-and-replace” strategies combining ORIF with total hip arthroplasty (THA) have emerged as an alternative to ORIF alone. This approach aims to achieve immediate stability and enable early mobilization, thereby minimizing the risks associated with prolonged immobilization in the elderly [8, 9]. In osteoporotic bone and in the presence of dome or marginal impaction, cementless cups may necessitate protected weight bearing; by contrast, cemented acetabular components can provide more predictable primary stability and may permit immediate full weight bearing, which is a key rationale for acute THA in this setting [10, 11].

Several series have reported favourable early outcomes when cemented acetabular components, including cemented dual-mobility cups, are used as part of these combined procedures in frail elderly patients with acetabular fractures [12, 13].

The aim of this study was to compare treatment outcomes between acute THA with open reduction and internal fixation using a cemented cup (C-THA) and ORIF alone in elderly patients with acetabular fractures. Based on concerns that ORIF alone and some cementless constructs may not provide sufficient primary stability for early full weight-bearing in osteoporotic bone, our primary hypothesis was that C-THA would provide stable immediate full weight-bearing without loss of reduction and thereby improve early functional recovery compared with ORIF alone, addressing a gap in the literature regarding postoperative stability in this vulnerable population.

## Materials and methods

### The study design and patient characteristics

This retrospective cohort study was conducted at a Level 1 trauma center (Department of Emergency and Critical Care Medicine, Saitama Medical Center, Saitama, Japan) and regional orthopaedic trauma center (Division of Orthopaedic trauma, Sapporo Tokushukai Hospital, Hokkaido, Japan), evaluating the efficacy of cemented total hip arthroplasty (C-THA) combined with open reduction and internal fixation (ORIF) in elderly patients with acetabular fractures. The study was conducted based on approval by the Ethics committee of Saitama Medical Center. From 2012 to 2022, 322 patients with acetabular fractures were treated at the two participating centers. For the present study, we included patients aged 60 years or older with displaced acetabular fractures who underwent operative treatment. We excluded patients managed nonoperatively, those with non-displaced or minimally displaced fractures (including those treated with percutaneous fixation only), those with concomitant ipsilateral lower limb injuries that precluded functional assessment, those with a follow-up period of less than 3 months, and those who were unable to complete the functional evaluation. After applying these criteria, 51 patients were eligible and formed the study cohort; they were divided into the C-THA group (*n* = 10) and the ORIF group (*n* = 41) for comparison.

Patient characteristics were recorded, including age, sex, Poor Prognostic Factors (PPFs), and follow-up period. Baseline characteristics and between-group comparisons are presented in Table 1. There were no significant differences in age and sex between the groups. On the other hand, Poor Prognostic Factors (PPFs) were significantly more frequent in the C-THA group, and the follow-up period was significantly longer in the ORIF group.

**Table 1:** Patient characteristics

Characteristics	Total	C-THA	ORIF	*p*-value
No. of the patients^*1^	51	10 (8/2)	41 (18/23)	
Age (Median [IQR])	69 [65–77.5.5]	72 [70–76]	67 [64–78]	0.216 ^*2^
Gender (M: F)	39:12	7:3	32:9	0.270 ^*3^
Poor Prognostic Factors (Median [IQR])	3 [1–4]	3.5 [3–4]	2 [1–3]	0.025 ^*2^
Follow up months (Median [IQR])	24 [10–49.5.5]	9.5 [6.3–13.8]	24 [12–65]	0.013 ^*2^

*1 Number of patient in (Saitama Medical Center/Sapporo Tokushukai Hospital)

*2 Mann-Whitneys U-test

*3 Fisher’s exact test

### Fracture classification

Fracture classification was determined based on standard radiographs and CT scans, according to the Judet-Letournel classification system [14]. Because classification was performed by a single rater, inter-observer reliability (κ) was not estimable; classification was therefore used to describe the cohort rather than to drive the primary comparative inferences (Table 2).

**Table 2:** Fracture type distribution according to Judet-Letournel classification

Fracture type	C-THA	ORIF	Total
Anterior column	1	7	8
Posterior column	0	1	1
Posterior wall	4	1	5
Transvers	1	2	3
Transvers + posterior wall	0	2	2
Posterior column + posterior wall	1	0	1
T shape	0	1	1
Anterior column + posterior hemi-transvers	2	14	16
Both column	1	13	14

In addition, we recognize that substantial morphological and severity variation may exist even within the same Judet–Letournel category (e.g., comminution, impaction, instability/dislocation patterns). Therefore, we complemented classification with predefined morphology-based poor prognostic factors (PPFs) to better characterize fracture complexity beyond classification labels.

### Indications and PPF definition

Indications and policy. Our treatment policy evolved as institutional experience accumulated. From 2021 onward, we favored acute cemented total hip arthroplasty (C-THA) when ≥ 3 poor prognostic factors (PPFs) were judged present on clinical and CT/radiographic assessment.

PPF definition (operationalized for this study). Guided by the geriatric acetabular framework described by Butterwick et al. (JBJS Am, 2015) [15], we operationalized PPFs as binary (present/absent) and restricted the set to the following seven items for this analysis:

(1) posterior wall comminution; (2) marginal impaction; (3) roof impaction; (4) hip dislocation (including posterior or central/medialization patterns); (5) femoral head impaction; (6) femoral head and neck fractures; and (7) preexisting arthritic change.

The selection flowchart reflecting this policy is provided in Table 3. These PPFs were selected to capture clinically relevant fracture morphology and joint/pathology features that are not fully reflected by fracture classification alone, thereby partially addressing within-category heterogeneity.()

**Table 3:**
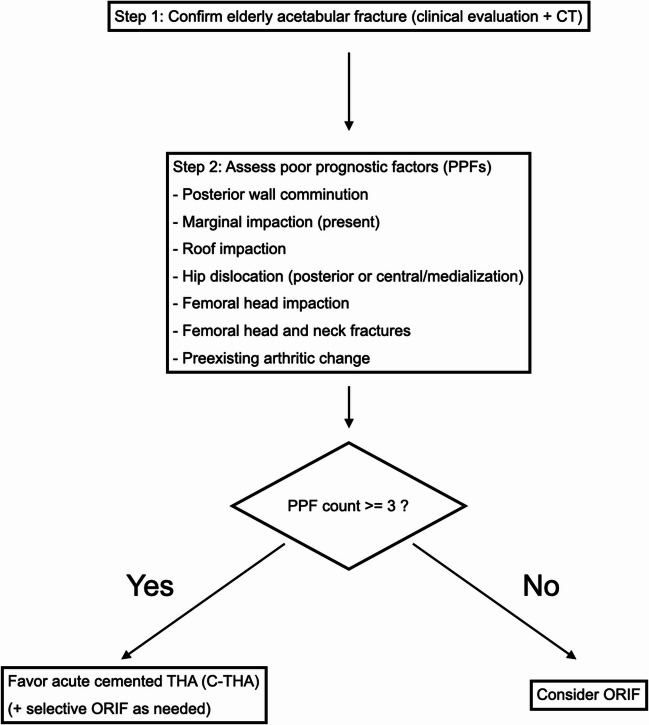
Treatment selection flowchart for elderly acetabular fractures (institutional policy since 2021)

The pathway favours acute cemented total hip arthroplasty (C-THA) when ≥ 3 predefined poor prognostic factors (PPFs) are present; otherwise, open reduction and internal fixation (ORIF) is considered. Exceptions (patient preference, contraindications, intra-operative findings) may override

*C-THA* cemented total hip arthroplasty, *ORIF* open reduction and internal fixation, *PPF* poor prognostic factor

### Surgical intervention

In both groups, the surgical technique and surgical approach were tailored to the fracture pattern as assessed on preoperative radiographs and CT. In the C-THA group, the procedure consisted of open reduction and internal fixation followed by cemented THA in the same sitting. Displaced anterior and/or posterior column fragments were first reduced and stabilized with plates and screws to restore the acetabular ring; cup implantation was performed only after the operating surgeon judged the reduction and construct to be stable on direct visualization and fluoroscopic images. When a defect of the acetabular weight-bearing dome was present, impaction bone grafting using morselized femoral head autograft was performed to reconstruct the dome and provide a continuous bed for cement. A low-viscosity polymethylmethacrylate cement was then pressurized into the prepared acetabulum, with particular care to avoid cement interposition within fracture lines; intraoperative fluoroscopy was used to confirm that cement penetration across fracture lines did not occur. The goal of this acute THA strategy was to provide sufficient construct stability to permit immediate full weight-bearing postoperatively at the discretion of the treating surgeon.

**Fig. 1:**
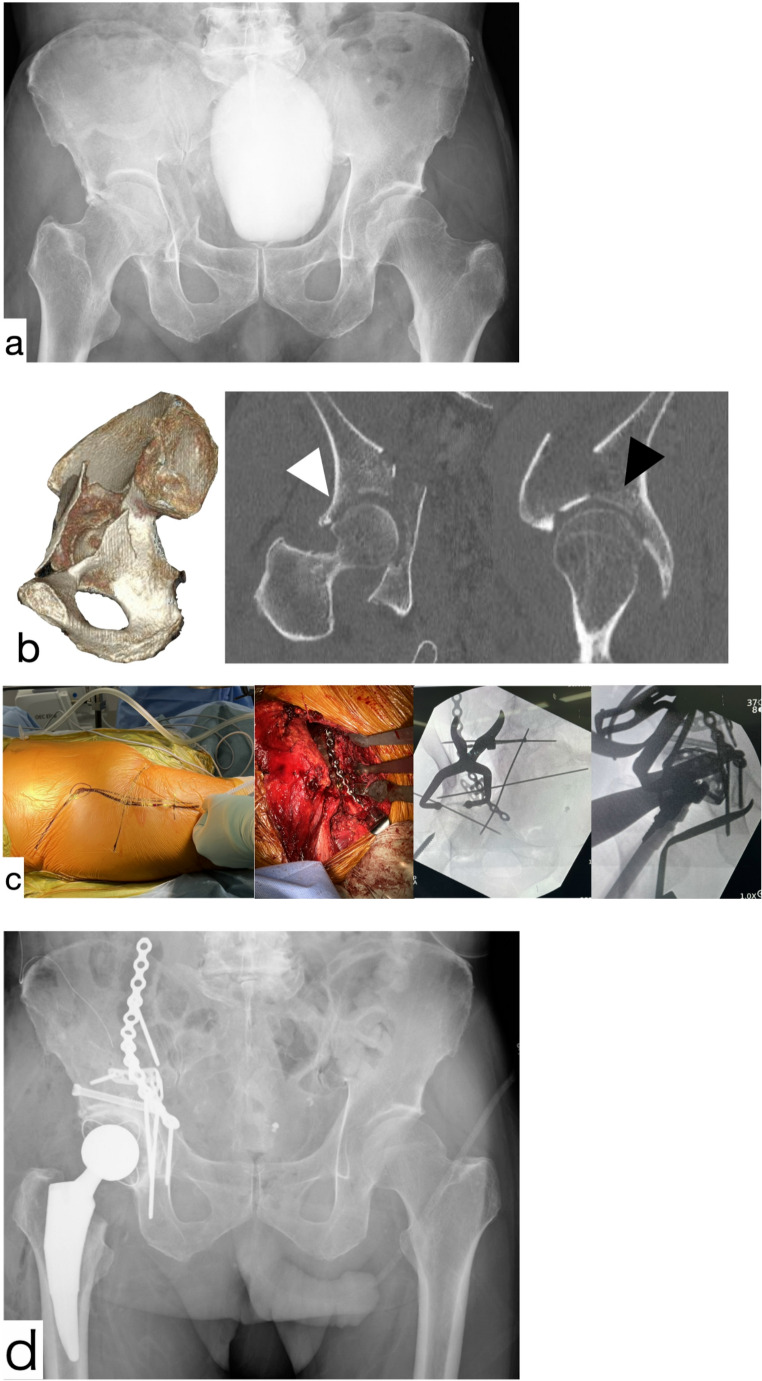
73-year-old male with anterior column fracture treated by C-THA using the iliofemoral approach AP X-ray at the time of injury. Dome impaction and femoral head injury existed CT scan at the time of injury. 3D CT shows the anterior column and isolated QLS fractures. Coronal view shows femoral head impaction (white arrow). The sagittal view shows a dome impaction fragment (black arrow) Intraoperative findings show skin incision, plate fixation through iliofemoral approach, and intraoperative image views AP X-ray just after the operation (C-THA). HHS at 3 months was 79

**Fig. 2:**
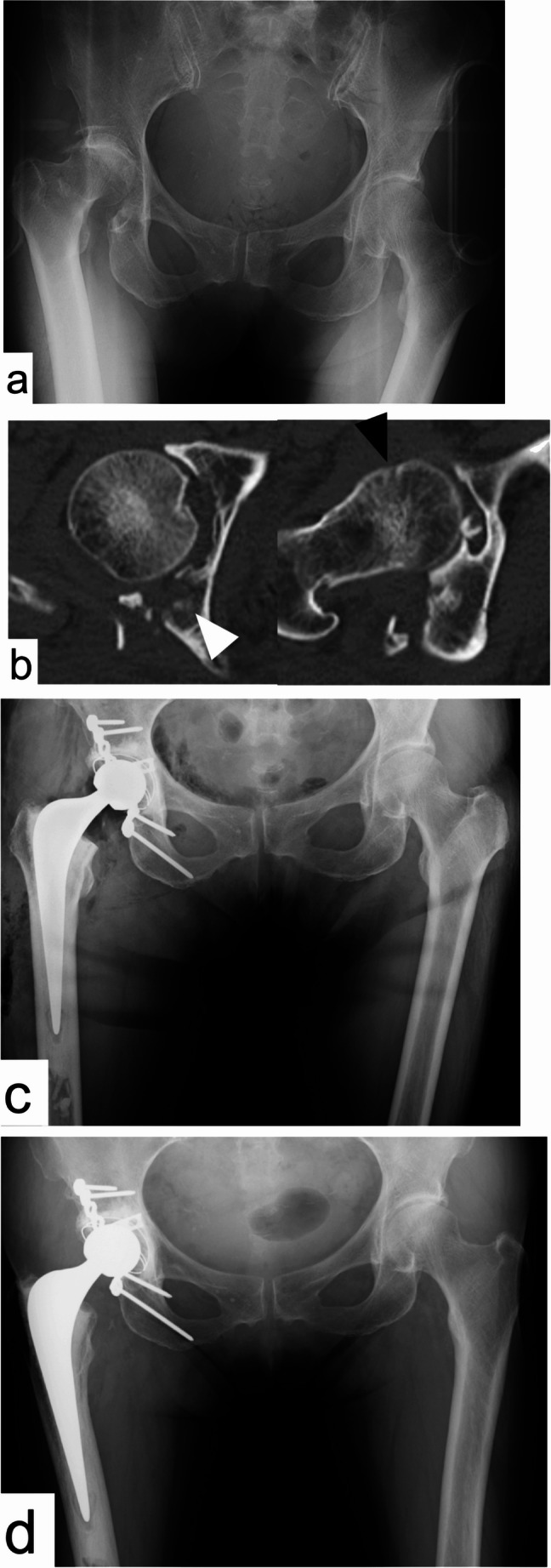
75-year-old female with posterior wall fracture and hip dislocation treated by C-THA using Kocher-Langenbech approach AP X-ray at the time of injury reveals right hip fracture dislocation CT axial view reveals marginal impaction (white arrow), posterior wall comminution, and femoral head impaction (black arrow). This case had four PPFs Postoperative AP X-ray. In this case, THA with cement cup was performed in addition to ORIF of posterior wall. HHS at 3 months was 82.9 Two-year postoperative AP X-ray

For anterior fracture patterns in the C-THA group, we used an iliofemoral approach for ORIF, followed by a direct anterior THA performed through a distal extension of the same incision (Fig. 1). For posterior fracture patterns, the Kocher–Langenbeck approach was employed for both ORIF and cup/stem implantation (Fig. 2). In this series, all C-THA procedures were completed through a single-incision approach.

**Fig. 3:**
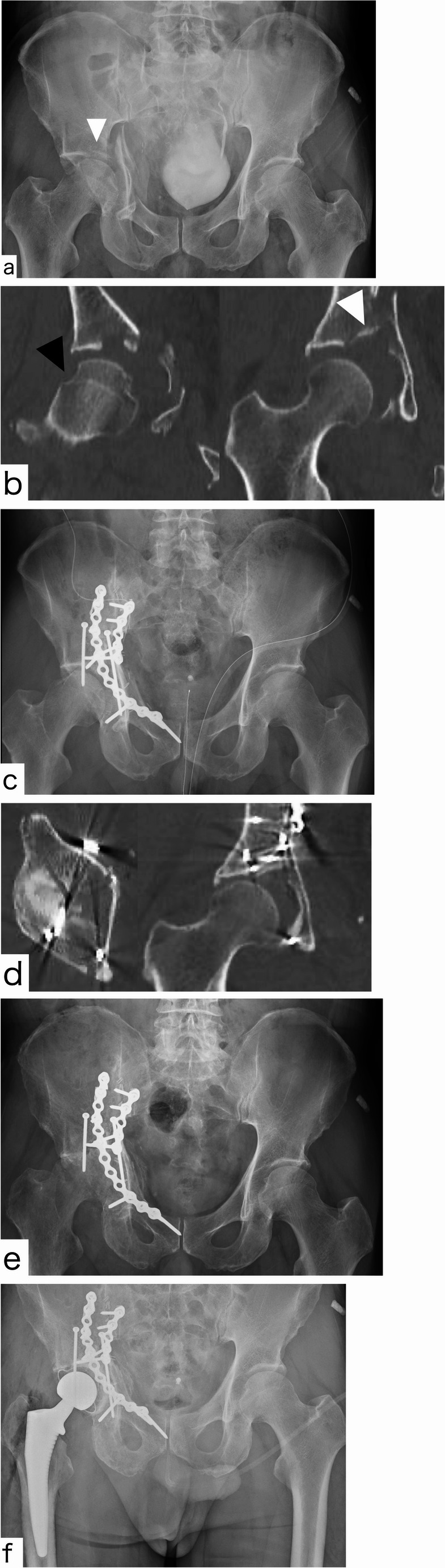
65-year-old male with anterior column and posterior hemitransvers fracture treated by ORIF using pararectus approach AP X-ray at the time of injury. Dome impaction and medialization of femoral head existed CT coronal views revealed femoral head impaction (black arrow) and dome impaction (white arrow) Postoperative AP X-ray Postoperative CT revealed poor reduction quality of impacted fragment AP X-ray at three months postoperatively shows end-stage hip arthritis. HHS at 3 months was 29.7 AP X-ray after the THA conversion

In the ORIF group, fracture fixation followed standard contemporary principles according to the fracture pattern. For anterior fracture patterns, either the modified Stoppa approach (with extension to the first window of the ilioinguinal approach when needed) or the pararectus approach was used. Reduction and fixation aimed to restore both columns and the quadrilateral surface using buttress plating along the iliopectineal line and quadrilateral surface as the primary method of fixation, with anterior column screws added when required to achieve sufficient stability. For posterior fracture patterns, a Kocher–Langenbeck approach was utilized (Fig. 3); posterior column fractures were treated with compression plating, and posterior wall fractures were stabilized with buttress plates.

Fracture union in both groups was assessed on serial radiographs by the presence of cortical and/or bridging trabeculation on pelvic AP, iliac oblique, and obturator oblique views, with CT obtained when indicated.

### Outcomes

Peri-operative data included operative time and intraoperative blood loss. Intraoperative blood loss (mL) was obtained from the anaesthesia record and calculated as the volume in suction canisters minus the volume of irrigation fluid, plus the weight difference of surgical sponges; postoperative drain output was not included.

Early functional outcome was assessed using the Harris Hip Score (HHS) at 3 months postoperatively. This time point was pre-specified as an “early recovery” window in this elderly fracture population, consistent with our hypothesis that acute C-THA would facilitate early mobilization and functional recovery. A 3-month HHS has also been used as an early functional endpoint in previous reports of acute THA for elderly acetabular fractures [16].

Peri-operative complications were defined a priori as surgical complications occurring within 30 days of the index procedure. Specifically, we recorded iatrogenic nerve injury, intraoperative vascular injury, surgical site infection, and postoperative loss of reduction.

For patients in the ORIF group, conversion to THA during follow-up was defined as a secondary THA performed for either (i) painful end-stage post-traumatic osteoarthritis or (ii) mechanical failure (loss of fixation or secondary displacement), in both situations with radiographic confirmation of structural failure or end-stage joint degeneration.

Continuous variables were summarised as medians with interquartile ranges (IQRs). Because most distributions were skewed and one treatment group was relatively small (C-THA *n* = 10), between-group comparisons for continuous outcomes were performed using the Mann–Whitney U test. For each continuous outcome, we additionally report the absolute between-group difference in medians (Δ) and the standardised mean difference (Hedges g) with 95% confidence intervals to emphasise estimation rather than dichotomous hypothesis testing.

Categorical variables were summarised as counts and percentages and compared between groups using Fisher’s exact test (or the χ² test when all expected cell counts were ≥ 5).

Given the limited sample size and the exploratory nature of several secondary comparisons, we did not apply a formal adjustment for multiple testing; p-values are therefore interpreted as descriptive, and we focus our interpretation on the magnitude and precision of the effect estimates. We considered multivariable modelling to adjust for potential confounders such as the higher prevalence of poor prognostic factors (PPFs) in the ORIF group, but the small overall sample and low number of events would have rendered such models statistically unstable and at high risk of overfitting. We therefore present unadjusted group comparisons and explicitly acknowledge the potential for residual confounding in the Discussion.

Because follow-up duration differed between groups (median [IQR] 9.5 [6.3–13.8] months in C-THA vs. 24 [12–65] months in ORIF), our primary comparative endpoints were early, fixed-window outcomes (time to full weight bearing, operative time, blood loss, and Harris Hip Score at 3 months). Time-dependent late outcomes (implant survival, postoperative osteoarthritis progression, and THA conversion) are reported descriptively without between-group statistical inference.

Given the low incidence and retrospective two-centre design (2012–2022), all consecutive eligible patients were included and no a priori sample-size calculation was feasible. To prioritise estimation over dichotomous testing, we report for continuous outcomes the between-group absolute difference in medians (Δ) and standardised mean difference (Hedges g). For skewed variables with medians/IQRs only, SDs were approximated from IQR/1.35, and Hedges g was computed using the pooled SD (with small-sample correction). Two-sided α = 0.05. All statistical analyses were performed using R software to ensure robust and reliable evaluation of the collected data [17].

## Results

Consistent with our institutional selection policy, the overall burden of morphology-based poor prognostic factors (PPFs) was higher in the C-THA group than in the ORIF group (median [IQR] 3.5 [3, 4] vs. 2 [1–3], *p* = 0.025). The C-THA group achieved significantly earlier full weight-bearing than the ORIF group (median 3.9 [IQR 2.8–5.0] vs. 70 [63–70] days, *p* < 0.001). Median operative time was also shorter in the C-THA group (228 [212–232] vs. 303 [239–355] minutes, *p* = 0.019). Blood loss tended to be lower with C-THA (median 914 [453–1292] vs. 1300 [863–1933] mL; Δ − 386 mL, ~ 30% lower; Hedges g ≈ − 0.50), although this difference did not reach statistical significance (*p* = 0.060). HHS at 3 months showed no statistically significant difference (median 79.9 [77.4–83.0] vs. 68.0 [44.9–82.0], *p* = 0.180), but the median difference favoured C-THA by + 11.9 points (~ 18% higher; Hedges g ≈ + 0.47, moderate) (Table 4).

**Table 4:** Comparison between C-THA and ORIF about study outcomes

Variable	C-THA	ORIF	*p*-value
FWB (days, Median [IQR])	3.9 [2.8–5.8]	70 [63–70]	< 0.001 ^༊1^
Operative time (min, Median [IQR])	228 [212–232]	303 [239–355]	0.019 ^༊1^
Blood loss (ml, Median [IQR])	914 [453–1292]	1300[863–1933]	0.060 ^༊1^
Bone union	10/10	41/41	NA
HHS at 3 months	79.9 [77.4–83.0]	68.0[44.9–82.0]	0.180 ^༊1^
Perioperative Complications	1/10 (10%)	11/41 (27%)	0.199 ^༊2^
Implant survival (%)	10/10 (100%)	NA	NA
OA change (%)	NA	18/41 (44%)	NA
THA conversion (%)	NA	10/41 (24%)	NA

༊1 Mann-Whitney U test

༊2 Fisher’s exact test

## Discussion

Our study reveals that C-THA combined with ORIF appears to enhance postoperative stability, enable immediate full weight-bearing, and facilitate early functional recovery in elderly patients with acetabular fractures. This approach has shown a marked improvement in short-term outcomes, notably in the speed of rehabilitation and reduction in immobilization-associated risks.

In our study, we compared outcomes between C-THA and ORIF groups. Since acetabular fractures are intra-articular, standard postoperative care following ORIF involves a period of non-weight-bearing. Therefore, it is natural that weight-bearing was initiated later in the ORIF group compared to the C-THA group, which allowed for immediate full weight-bearing. However, in acute THA using non-cemented cups, effective press-fit fixation is often unachievable due to the presence of fractures in the acetabulum, necessitating supplemental screw fixation. Even with screws, studies have reported that a certain period of protected weight-bearing is still required [10, 18, 19]. There is also a report of an immediate full weight bearing using a large non-cemented cup, but it is reported that there have been cases of loosening in the postoperative course, which raises concerns about implants and placement when the goal is to achieve an immediate postoperative FWB [20]. Consequently, using a cemented cup may offer a significant advantage over both ORIF and non-cemented acute THA in terms of postoperative rehabilitation. The non-significant HHS at 3 months difference likely reflects a combination of limited power (C-THA *n* = 10) and the short assessment window (3 months), which may not fully capture functional convergence/divergence after rehabilitation. The observed effect estimate (≈ + 12 points) is clinically directional and aligned with immediate FWB, but longer and balanced follow-up is required to determine whether early gains persist or widen.

Our study also revealed that the C-THA group had significantly shorter operative times and a trend toward less intraoperative blood loss compared to the ORIF group. Logically, one might expect that acute THA, which involves both fracture fixation and arthroplasty, would result in longer operative times and greater blood loss [21]. However, acetabular fractures in elderly patients frequently involve dome impaction, marginal impaction, and comminuted fragments. While ORIF requires substantial time to anatomically reduce these comminuted fragments, our C-THA approach focuses on reducing the main columns without attempting to reduce fragmented portions of the acetabular roof. As a result, the time otherwise spent addressing the joint surface and comminuted bone fragments can be spared. Moreover, since the acetabular columns are reconstructed, cup implantation can be performed into a reconstructed, near-anatomic acetabulum, reducing technical difficulty. This strategy appears to contribute to reduced surgical invasiveness overall, as reflected in operative time and blood loss.

A notable aspect of our study is that poor prognostic factors (PPFs) were significantly more common in the C-THA group (*p* = 0.025, Table 1). This finding underscores the complexity of decision-making in treating elderly patients with acetabular fractures. The presence of PPFs could potentially guide the choice between ORIF and THA during the preoperative planning phase. A systematic review suggests that elderly acetabular fracture patients with several prognostic factors are ideal candidates for acute THA due to the high rate of conversion to THA following failed ORIF [22]. Our findings support the notion that identifying patients with significant PPFs may indicate higher suitability for C-THA, considering its benefits of immediate weight-bearing and accelerated recovery. This approach is consistent with the individualized treatment strategy advocated by Boraiah et al. and Herscovici et al., emphasizing the importance of personalized patient assessment to achieve optimal outcomes [6, 8].

The anterior column with posterior hemitransverse pattern with quadrilateral surface medialization—frequently encountered in older adults—illustrates this case-selection challenge. In our experience, this pattern can still achieve satisfactory outcomes with ORIF alone when the overall burden of poor prognostic factors (PPFs) is limited and stable anterior column reconstruction with quadrilateral buttressing is achievable. Conversely, irrespective of the fracture pattern, patients with multiple PPFs are at higher risk of failure or early conversion after ORIF alone; in such cases, combining ORIF with acute C-THA may be the more appropriate strategy to enable reliable early mobilization and reduce the likelihood of secondary procedures.

Because our institutional policy changed in 2021 to prefer C-THA when patients had ≥ 3 PPFs, treatment allocation was not random. Patients who underwent C-THA generally had more risk factors. To avoid over-interpretation, we focus on short-term safety and the feasibility of early mobilization, rather than claiming long-term superiority. Any similar or better short-term outcomes in the C-THA group should be regarded as conservative, given their higher baseline risk. In addition, treatment selection inevitably reflected surgeon judgment (including perceived stability, bone quality, and anticipated ability to comply with weight-bearing restrictions) and evolving institutional experience over the study period, which introduces confounding by indication. Given the small sample size, we could not perform robust risk-adjusted analyses (e.g., propensity methods or multivariable modelling) without a high risk of overfitting; therefore, our comparisons should be interpreted as descriptive rather than causal estimates.

In addition to patient-related prognostic factors, the choice of implant and fixation method is another critical determinant in the success of acute THA. Our study specifically evaluated the use of cemented cups in C-THA, which remains relatively underexplored in the literature. A major concern regarding cemented total hip arthroplasty has historically been its long-term outcomes, primarily due to the risk of cup loosening over time. In earlier foundational work, Mears et al. adopted a strategy involving cementless cups with postoperative weight-bearing restrictions to mitigate this issue in the context of acetabular fractures [4].

In contrast, our study employed cemented cups in all cases, demonstrating favorable short- to mid-term clinical outcomes, including implant stability and early mobilization. These findings are aligned with recent studies that, although limited by relatively short observation periods, have reported satisfactory implant survival rates when cemented acetabular components were used [23, 24]. These results collectively support the rationale for considering cemented cups as a first-line option in acute THA for elderly patients with acetabular fractures, especially when early full weight bearing and functional recovery are desired.

Large-scale registry data from the Nordic Arthroplasty Register Association (NARA) report high 10-year survivorship of cemented THA in older patients (≈ 94–96%) [23]. Reverse-hybrid combinations (cemented cups with uncemented stems) show a slightly higher revision risk than fully cemented constructs, although absolute survivorship remains high (around 92% at 10 years) in these registries [23]. These findings suggest that cemented acetabular fixation is durable in the elderly, supporting our rationale for enabling immediate FWB. However, most registry evidence is not fracture-specific; in the acetabular-fracture setting, early mid-term series (including cemented dual-mobility constructs) report encouraging stability and low early failure, yet long-term data remain limited [25]. We therefore interpret our favourable early results with caution and stress the need for extended follow-up focused on aseptic loosening, particularly in cases with dome defects where impaction bone grafting and a uniform cement mantle are critical for longevity (27).

Our study’s insights are drawn from observations across two institutions, yet we recognize several limitations. The lack of long-term follow-up restricts our understanding of the sustained outcomes and potential late complications of cemented cup THA. Although data from two institutions enhance the diversity of our sample, the overall number of cases remains limited, affecting the generalizability of our conclusions. Treatment was delivered across two institutions with potentially different expertise, evolving protocols, and learning curves, introducing possible operator or institutional bias that may influence both treatment selection and outcomes.

Fracture types were assigned by one surgeon, precluding an inter-observer κ estimate; however, our main inferences rely on predefined PPFs and early, fixed-window outcomes rather than granular subtype comparisons, which limits the potential impact of classification variability. More importantly, substantial fracture heterogeneity exists even within the same Judet–Letournel category (e.g., comminution, impaction, instability/dislocation patterns, and quadrilateral surface medialization), which may influence both treatment selection and outcomes. Although we used predefined morphology-based PPFs to better characterize this complexity beyond classification labels, residual heterogeneity remains and limits the generalizability of between-group comparisons.

The C-THA group had a shorter follow-up than the ORIF group (median 9.5 vs. 24 months). This asymmetry may underestimate late complications or failures in C-THA (e.g., aseptic loosening), while inflating cumulative events in ORIF (e.g., OA progression, conversions) simply because of longer observation time. To minimize this bias, we predefined early, fixed-window outcomes (t-FWB, operative time, blood loss, HHS at 3 months) as the main comparative endpoints and presented late outcomes descriptively only. A longer, balanced follow-up is required to draw firm conclusions about long-term superiority.

Because this is a retrospective cohort of a low-incidence injury, the overall sample—particularly the C-THA group—limits precision and power for modest effects. Consequently, non-significant results (e.g., blood loss, HHS at 3 months) should be interpreted in light of the effect sizes, which indicate moderate, clinically directional differences but likely with wide confidence intervals under the present sample size.

We also acknowledge that important frailty-related baseline variables (e.g., BMI, ASA class, living situation, and detailed comorbidities) were not systematically recorded throughout the study period and could not be analysed, so residual confounding by patient health status and frailty cannot be excluded. Older patients are a heterogeneous population; pre-injury functional status, activity demand, cognitive function, and bone quality/osteoporosis may differ substantially even within the same age range and can materially affect both recovery trajectories and the risk–benefit balance between ORIF and acute THA. Because these domains were not consistently captured in this retrospective dataset, we could not adjust for them, and some degree of residual confounding by baseline function and physiological reserve is likely.

Finally, the external validity of our findings may be constrained by differences in healthcare systems and reimbursement policies across countries; in some settings, acute THA for acetabular fractures may not be routinely supported or financially feasible in the acute phase. Therefore, our results should be interpreted primarily as evidence informing case selection and the potential benefits of a combined reconstruction-and-arthroplasty strategy, rather than as an immediately generalizable care pathway across all healthcare environments.

In particular, prospective studies should incorporate standardized assessments of frailty and baseline function (e.g., pre-injury mobility/independence), as well as objective measures of bone quality/osteoporosis, to enable risk-adjusted comparisons and more generalizable case-selection guidance. Future research should aim for larger, multicenter studies with longer follow-up durations to more definitively establish the role of C-THA in the treatment of elderly patients with acetabular fractures.

## Conclusion

In conclusion, our study suggests that C-THA may be an effective method for achieving early functional recovery in elderly patients with displaced acetabular fractures. The immediate postoperative benefits, including enhanced stability and the capacity for early weight-bearing, highlight C-THA’s potential in acute treatment settings. Nonetheless, there is a pressing need for further research, especially studies that include long-term follow-up, to validate these findings and explore the long-term implications of C-THA for acetabular fractures in the elderly.

## Data Availability

No datasets were generated or analysed during the current study.
